# Mycorrhizal fungi and *Pseudomonas fluorescens* application reduces root-knot nematode (*Meloidogyne javanica*) infestation in eggplant

**DOI:** 10.1016/j.sjbs.2021.05.054

**Published:** 2021-06-01

**Authors:** Meenakshi Sharma, Ishan Saini, Prashant Kaushik, Mona Mohammed Aldawsari, Thamer Al Balawi, Pravej Alam

**Affiliations:** a Department of Botany, Kurukshetra University, Kurukshetra 136118, Haryana, India; b Instituto de Conservación y Mejora de la Agrodiversidad Valenciana, Universitat Politècnica de València, 46022 Valencia, Spain; c Department of Biology, College of Science and Humanities, Prince Sattam bin Abdulaziz University, Al-Kharj 11942, Saudi Arabia

**Keywords:** Arbuscular mycorrhizal fungi, Nematodes, Eggplant, Morphometrics, Antioxidant

## Abstract

Eggplant cultivation is subjected to attacks by numbers of pests and diseases from the nursery stage until harvest. Root-knot nematode (*M. javanica*) is one of the most significant restrictions in the successful cultivation of eggplant as it damages the crop year-round. One of the most essential classes of plant symbionts is arbuscular mycorrhizal fungi (AMF) and phosphate solubilizing bacteria (PSB), which significantly impact plant development, feeding, disease tolerance, and resistance to *M. javanica*. Eggplant seedlings were inoculated with two mycorrhizal fungi, Glomus mosseae (Gm) and Gigaspora gigantea (Gg), together with the phosphate-solubilizing bacteria (PSB) *Pseudomonas fluorescens* (Pf; ATCC-17400) under the presence of nematodes inoculation of *Meloidogyne javanica a*s 1000 eggs of *M. javanica* in each pot. Observations were recorded for 9 morphological traits, 6 fruit morphometric traits using Tomato Analyzer (version 4) software program, and 4 fruit biochemical traits. Along with the data recorded for mycorrhization (%), number of galls and reaction to RKN. Plants inoculated with the consortium (Pf + Gm + Gg) performed substantially better for most traits. Furthermore, the eggplant plants treated with consortium developed the highest levels of fruit biochemical content along with the highest level of mycorrhization (68.20%). Except for certain fruit morphometric traits, the treatment containing Pf + Gg outperformed the treatment containing Pf + Gm. Overall, this research showed that AM fungi could be a sustainable solution to the eggplant RKN problem.

## 1 Introduction

Eggplant is an important vegetable crop, well established for cultivation in Asia, Africa, America, and Europe (Taher et al., 2017). Eggplant fruit comes in various shapes, sizes, and colors, and is highly rich in vitamins and other beneficial nutrients like chlorogenic acid (Kaushik et al., 2018, Kaushik et al., 2015). However, its cultivation faces severe challenges from various insect pests and associated diseases. One among its wide-ranging pests is the root-knot nematode (RKN), which causes stunting of the plants and a noticeable reduction in yield (Rao and Kumar, 2017, Zhou et al., 2018). Among the several ways to control RKN infestation, it is more common to use chemical nematicides. To the extent that they are synthetic, these substances are harmful to the ecosystem, and do not ensure environmental protection (Hajek and Eilenberg, 2018). Further, among the various nematode species known for affecting the eggplant, infestation by RKN results in severe yield losses (Ralmi et al., 2016), mainly due to its broad host range shackling the accessibility of resistant/immune crops (Rashidifard et al., 2018, Kaur et al., 2018).

Several approaches have been tried to control the RKN infestation in eggplant, including breeding methods that use wild relatives of eggplant, such as *S. torvum*, for their high resistance to *Meloidogyne* spp. (Daunay et al., 2019, Saini and Kaushik, 2019). Also, eggplants have been genetically modified using the Mi-1.2 gene, thereby developing *M. javanica* tolerance (Barbary et al., 2015). Papolu et al. (2016) proposed that cystatins may help enhance eggplant tolerance in a technique that might improve crop yield (Coyne et al., 2018, Talwana et al., 2016). Further, whereas break-in nutrition supply or inadequate mineral nutrition, with RKN infestation, could lead to plants' death (Veronico et al., 2018), the application of biofertilizers not only enhances plant growth and development but also useful for pest control (Khanna et al., 2019a, Khanna et al., 2019b, Khanna et al., 2019c, Khanna et al., 2019d, Khanna et al., 2019e, Sharma et al., 2020). Manures have also been confirmed to suppress nematode pests in numerous studies worldwide (Öçal et al., 2018, Sulaiman and Mohamad, 2020).

Besides, the microbes such as Endomycorrhizal fungus, with *Pseudomonas fluorescens* have been widely studied to protect and improve plant quality and yields (Kumar et al., 2015, Latef et al., 2016, Drobek et al., 2019;). Colonization of arbuscular mycorrhizal fungus (AMF) is considered the key factor in maintaining plant-species for the stability of ecosystems. Their application leads to plant development through adjustment of phytohormones (Begum et al., 2019a, Malhi et al., 2021). Further, AMF possess a strong capacity to improve translocation and absorption of vital nutrients through fungal hyphae in vegetables. These bio-inoculants increase the accessibility of soil-enriching nutrients such as P, N, Fe and K. Phosphorus is essential for the production of nuclear acid (Begum et al., 2019b, Arora et al., 2020). Therefore, the plant benefits from AMF due to increased nutrient and water absorption in addition to the shielding of roots from soil-borne diseases (Priyadharsini and Muthukumar, 2015, Kumar et al., 2015, Latef et al., 2016, Ranganathswamy et al., 2019). It is a well-known fact that RKN and AMF are frequent co-inhabitants of vegetation origins and are mutually inhibitory (Rovenich et al., 2014). The greater tolerance of mycorrhizal treated plant life to comprehensive soil fungal, bacterial and nematodes has been proven (Duc, 2017).

Generally, AMF exhibit an antagonistic effect on plant-parasitic nematodes, and many studies have demonstrated a strong decline in nematodes as a result of the presence of healthy mycorrhizal density in the plants rhizosphere (Gough et al., 2020, Heinen et al., 2018, Poveda et al., 2020). *Pseudomonas* spp. is also a promising alternative for chemical fertilizers, and promotes the activity of AMF. *Pseudomonas fluorescens* grow well in mineral salts, accompanied by one or several carbon sources (Chakravarty and Anderson, 2015). Phosphate solubilizing bacteria (PSB) as a solution for the phosphate compounds, release organic acids into the soil available for use by plants. Since they are well suited in soil, *P. fluorescens* strains are thoroughly researched for their applications, including bacteria release and soil survival. This includes diseases, biocontrol in cultivation and bioremediation of different organic compounds (Kour et al., 2020, Qessaoui et al., 2019). Therefore, in this work, influence of mycorrhizal fungi and *Pseudomonas fluorescens* (ATCC-17400) on *M. javanica* and essential traits of eggplant were studied.

## 2 Materials and methods

### 2.1 Biological material and experimental details

In the experiment, the eggplant cultivar black beauty was used. Seeds were sown in plastic trays with a medium of three parts peat moss, one part perlite, one part vermiculite, and two parts of sterile soil in Botany Department, Kurukshetra University, Kurukshetra in 2017–2018 under regulated temperatures of 25–30 °C, 16-H photoperiod light of 8000 lx (in addition to natural sunlight), and 65–70 relative humidity. The plants were planted individually into 5 L plastic pots, and arranged with the same soil mixture in different containers. Plants were grown in randomized complete block design (RCBD) inside a greenhouse. Package and practices followed were defined elsewhere (Kaur et al., 2004).

### 2.2 AMF and PSB inoculation

*Glomus mosseae* inoculum containing 80–86% (w/w), *Gigaspora gigantea* inoculum containing 75–79% (root), and 870–890 AM spores (w/w) contained in Botany Department, Kurukshetra University, Kurukshetra, India. Dominant spores (at least 10,000 nos.) along with infected root parts, were utilized as bio-inoculum after mass production on maize as defined elsewhere (Saini et al., 2019). Any single treatment pot was filled with 100 g of each inoculum, and consortium treatment was applied to 50 + 50 g (*G. mosseae* + *G. gigantea*). The same AMF treatment dosage was repeated precisely 1 month after transplanting of plants. *P. fluorescens* (ATCC-17400) was procured from CSIR-Institute of Microbial Technology (CSIR-IMTECH) in Chandigarh. It was then maintained on a nutrient broth medium containing beef extract: 3 g/L; peptone: 5 g/L and NaCl: 5 g/L, respectively and incubated at 32 °C for 48 h. *P. fluorescens* was applied by dipping the roots for 10 min at the time of transplantation (Saini et al., 2020). There were 5 treatments as defined in Table 1.

**Table 1:** Treatments used in the present investigations.

Treatments	Code
Control (C)	T1
Normal Package (NP)	T2
*Pseudomonas fluorescens* (Pf) + *Glomus mosseae* (Gm) + NP	T3
Pf + *Gigaspora gigantea* (Gg) + NP	T4
Pf + Gm + Gg + NP	T5

### 2.3 Nematode penetration experiment

Eggplant plants were randomly removed from the nematode infested field and brought back to the laboratory. Fuchsin lactophenol acid treatment was then used for root staining for 2 h. To verify *M. javanica*, 5–10 mature females were separated from these roots using needles and forceps, teased with the stereoscopic binocular microscope to create perineal patterns for identification and confirmation of the *M. javanica* species. Single egg mass progeny was raised in pots when eggplants formed. *M. javanica* inoculum was harvested from the infected roots. Using forceps, tiny amounts of water and egg masses were separated from their Petri-plate and nematode extraction bases with Baermann's funnel technique (Christie and Perry, 1951). Sandy loam textured soil that belonged to the surrounding area, was sterilized in an autoclave for 15 lbs (121 ± 1 °C). The dried soil, was then filled in pots around 15 cm in diameter (1 kg capacity). For nematode-penetration experiment, 1 kg of steam sterilized soil was preserved in each pot. Each procedure was replicated three times under screen conditions. Every inoculated 1000 eggs and J2 *M. javanica*. According to crop specifications, pots were watered and aftercare was taken as required. The plants were rooted to record *M. javanica* volume of bile after 45 days of inoculation. The reactions were rated as defined by Gaur et al. (Table 2). Results were made on gall, numerous eggs and J2 per root system, and nematodes per 200 cc of soil. The roots were carefully collected and stored in a water pot to free it from soil pollutants. Roots were dispersed around the big Petri plate with water, for egg and J2 observations, and the final nematode population at 45 DAI was estimated. The galls were recorded after the seedlings were thoroughly cleaned and counted with the help of Hand convex. After proper teasing of gallic tissue, eggs and J2 were counted. Gallic tissue remains were isolated and finalized. Dilution processes estimated egg and J2 (Xing and Westphal, 2012).

**Table 2:** Root-knot scale for categorization of germplasm (Gaur et al., 2001).

Number of Galls	Nematode Reaction Scale
0	Highly Resistant
1–10	Resistant
11–30	Moderately Resistant
31–100	Susceptible
101 and above	Highly Susceptible

### 2.4 Plant morphological and fruit morphometric analysis

EGGNET descriptors were used to classify the plants morphologically (van der Weerden and Barendse, 2006). At flowering, Corolla Diameter (mm) was measured as mean of 5 flowers in every plant in each replication. Whereas Number of Flowers per Inflorescence were calculated at the time of flowering as an average of 3 inflorescence of each plant in every replication, Plant Height (cm) and Stem Diameter (mm) were recorded as one reading per plant. Leaf related characters, i.e, Leaf Pedicel Length (cm), Leaf Blade Length (cm), and Leaf Blade Width (cm) were taken as the average of 5 leaves per plant in each replication. Fruit Weight (g) was recorded as the average weight of 5 fruits in every replication and was expressed in grams (g). Except for Plant Height and Stem Diameter, which only allows for one measurement per plant, the remaining characters need at least three measurements per plant. Six fruit morphometric descriptors were analyzed with the help of Tomato Analyzer version 4 software program (Rodríguez et al., 2010): Three fruits per replicate were harvested at a commercial-stage (i.e., physiologically immature) and cut-opened longitudinally and scanned using an HP Scanjet Scanner (Hewlett-Packard, USA) at a resolution of 300 dpi. Traits recorded were Curved Fruit Shape Index, Perimeter (cm), Fruit Shape Triangle, Area (cm^2^), both fruit shape index external, i.e. I and II, Fruit Shape Triangle (cm), and Fruit Shape Index Internal, respectively.

### 2.5 Biochemical analysis

For the dry matter (%) estimation, 50 g of fresh fruit sample was dried in a hot air oven, and the percentage of change in weight before and after drying was recorded as dry matter content (%). Total sugars (%) content was estimated using 0.5 g of dried, powdered eggplant samples, further mixed with 20–25 mg of ethanol and heated in the water bath for 2 h. It was purified, and 5 ml acetate from lead was applied to the filtrate and maintained for 30 min, followed by addition of sodium oxalate. A 0.5 ml sample from this extract was taken and then diluted with 5 ml of aliquot, further 1 ml of purified water, 1 ml of phenol (5%) and 4 ml of H_2_SO_4_ were added. It was shaken and subsequently cooled to natural temperature, and recorded using an electronic spectrophotometer at 490 nm optical density. The total phenols (mg/100 g) were determined using volume of the extract, 0.5 ml (V1) was piped into a flask of 25 ml with 6–7 ml of water, followed by addition of a 0.5 ml Folin-Ciocalteu (double-diluted) regent sample. Just after 3 min, 1 ml of sodium carbonate was added, further purified water was added to make the volume to 25 ml(V). The optical density value was read at 760 nm after using water and sodium carbonate as blank for 1-hour (Luthria et al., 2010). Anthocyanin (mg/100 g) content was estimated as proposed by (Nothmann et al., 1976). Accordingly, 0.1 g(w) of fresh peel sample was taken and sliced into small pieces. Extraction was carried out by retaining it in 10 ml 1% hydrochloric acid (methanol) for two days in the refrigerator at low temperatures. The supernatant was purified, and solution optical density (A) read at 530 nm and 667 nm wavelength (for chlorophyll interference) using the formula defined in the original method.

## 3 Results

### 3.1 Effect on fruit morphometric traits

**Fig. 1:**
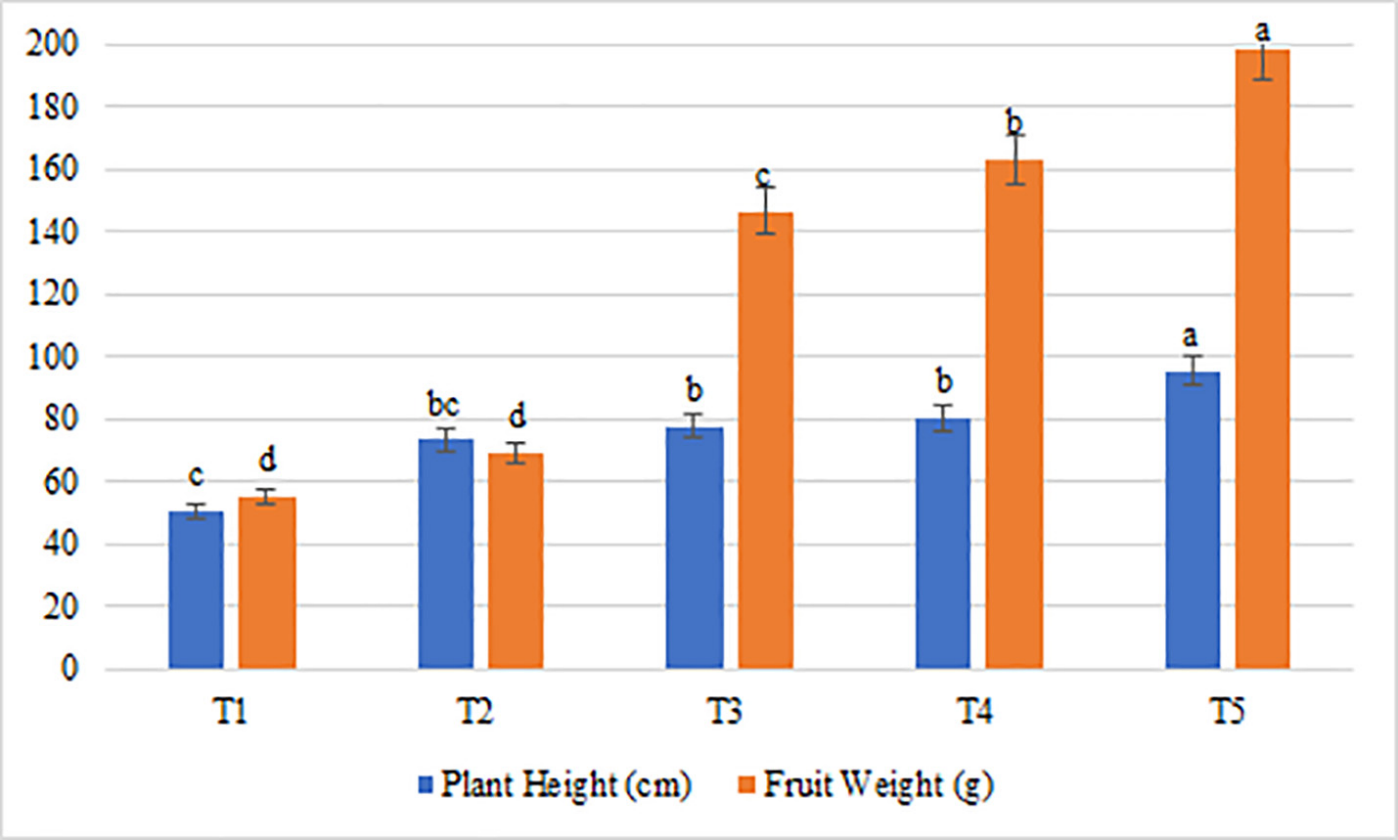
Variation recorded for the fruit weight (g) and plant height (cm) in the 5 treatments studied for eggplant under root-knot nematode (*M. javanica*) infestation.

The treatments with AMF and PSB produced significant effects in the morphological traits of eggplant (Table 3). Although the treatments with Pf + Gm + NP, (T3) and Pf + Gg + NP, (T4) demonstrated a considerable improvement in different traits, Pf + Gm + Gg + NP, (T5) was found to be the most efficient treatment (Table 3). Whereas, introduction of Pf + Gm and Pf + Gg increased the corolla diameter by 44.9% and 76.71% respectively, Pf + Gm + Gg showed a dramatic increase of 118.7% (Table 3). Likewise, leaf blade length exhibited an increase of 51.17%, 74.34% and 98.39% after application of Pf + Gm, Pf + Gg, and Pf + Gm + Gg, respectively, and leaf blade width increased by 46.29%, 60.18% and 103.7% when treated with Pf + Gm, Pf + Gg, and Pf + Gm + Gg, respectively (Table 3). Further, leaf pedicel length recorded an increase of 47.22% and 72.48% with Pf + Gm and Pf + Gg respectively, besides, it produced a remarkable increase of 129.36% with Pf + Gm + Gg, which again proved T5 to be the most effective against *M. javanica* infestation (Table 3). Plant height also increased by 53.2%, 58.7%, and 88.1% under the influence of Pf + Gm, Pf + Gg, and Pf + Gm + Gg, respectively (Fig. 1). In addition, the role of AMF and PSB in plant development was further justified with the increments of 125.5%, 150.3%, and 225.5% in the number of flowers per inflorescence when treated with Pf + Gm, Pf + Gg, and Pf + Gm + Gg respectively (Table 3). Besides, stem diameter increased by 35.2%, 44.6%, and 65.7% after introducing Pf + Gm, Pf + Gg, and Pf + Gm + Gg respectively, to the eggplant. However, it should be noted that the most significant results were produced in the fruit weight with increase of 165.7%, 195.7%, and 259.7% with Pf + Gm, Pf + Gg, and Pf + Gm + Gg, respectively, owing to the mycorrhizal activity of Gm and Gg along with phosphate solubilizing activity of Pf.

**Table 3:** The response of morphological traits of Eggplant to AMF and PSB application treatments under *M. javanica* infestation.

Traits	T1(C)	T2 (NP)	T3 (Pf + Gm)	T4 (Pf + Gg)	T5 (Pf + Gm + Gg)
Corolla Diameter (mm)	19.37d‡	22.33d	28.07c	34.23b	42.37a
Leaf Blade Length (cm)	14.97e	21.03d	22.63c	26.10b	29.70a
Leaf Blade Width (cm)	10.80d	14.37c	15.80bc	17.30b	22.00a
Leaf Pedicel Length (cm)	4.87e	6.03d	7.17c	8.40b	11.17a
Number of Flowers per Inflorescence	1.33c	3.01b	3.00b	3.33b	4.33a
Stem Diameter (mm)	12.67c	16.67b	17.13b	18.33b	21.00a

‡ Values in a column followed by the same letter are not significantly different, p ≤ 0.05, LSD.

### 3.2 Effect on agronomical, biochemical traits and nematode infestation

**Fig. 2:**
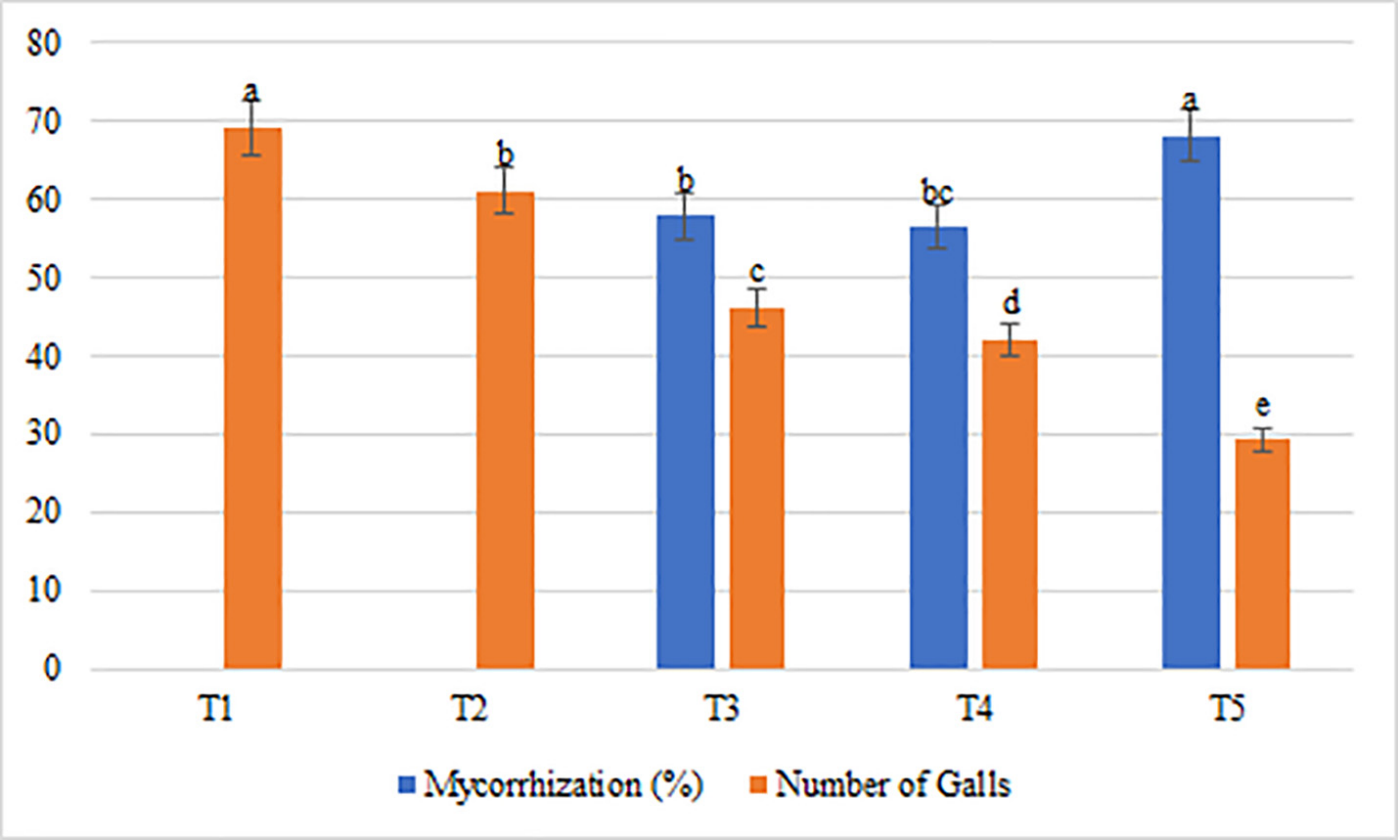
Variation recorded for the mycorrhization (%) and number of galls in the 5 treatments studied for eggplant under root-knot nematode (*M. javanica*) infestation.

Further, the variations in morphometric and biochemical traits of bell pepper under the influence of AMF (Gm and Gg), and PSB (Pf) were recorded in Table 4. It was observed that the perimeter increased by 79.7%, 127.4%, and 145.64% when treated with Pf + Gm, Pf + Gg, and Pf + Gm + Gg respectively (Table 4), while, the area increased drastically by 186.6%, 334.05%, and 419.8% respectively. In addition, fruit shape index external I and II increased only by 30.9% and 36.5% respectively after application of Pf + Gm; 82.5% and 104.3% respectively with Pf + Gg; but 160.8% and 204.3% respectively with Pf + Gm + Gg. Similarly, the curved fruit shape index increased by 37%, 107%, and 190% after the introduction of Pf + Gm, Pf + Gg, and Pf + Gm + Gg respectively (Table 4). In case of fruit shape index internal, increments of 36.5%, 107.5%, and 204.3% were observed when the plant was treated with Pf + Gm, Pf + Gg, and Pf + Gm + Gg respectively. However, the percentage of dry matter did not show any significant variations, it increased only marginally when treated with Pf + Gm (1.72%), Pf + Gg (1.81%), and Pf + Gm + Gg (3.93%) (Table 4). Similarly, no significant increase was observed in the percentage of total sugars, which was only 0.4% with both Pf + Gm and Pf + Gg, and 0.87% with Pf + Gm + Gg. The amounts of total phenols also increased by 11.87%, 13.72%, and 46.77% only after treatment with Pf + Gm, Pf + Gg, and Pf + Gm + Gg respectively (Table 4). However, the anthocyanin content recorded dramatic increase of 233.3% with both treatments Pf + Gm and Pf + Gg; and 476.6% with treatment Pf + Gm + Gg (Table 4). Furthermore, the effects of symbiotic association with AMF and PSB were found to be associated with increased mycorrhization by 57.9%, 56.42%, and 68.20% with Pf + Gm, Pf + Gg, and Pf + Gm + Gg respectively (Table 4, Fig. 2), and decreased number of galls by 33.26%, 39.04%, and 57.73% after treatment with Pf + Gm, Pf + Gg, and Pf + Gm + Gg respectively (Table 4, Fig. 2).

**Table 4:** The response of morphometric and biochemical traits of bell pepper to AMF and PSB under *M. javanica* infestation.

Traits	T1 (C)	T2 (NP)	T3 (Pf + Gm)	T4 (Pf + Gg)	T5 (Pf + Gm + Gg)
Perimeter (cm)	16.41e‡	21.03d	29.50c	37.33b	40.31a
Area (cm^2^)	17.47e	28.03d	50.07c	75.83b	90.81a
Fruit Shape Index External I	0.97d	1.07 cd	1.27c	1.77b	2.53a
Fruit Shape Index External II	0.93d	1.07 cd	1.27c	1.90b	2.83a
Curved Fruit Shape Index	1.00d	1.13d	1.37c	2.07b	2.90a
Fruit ShapeTriangle	0.61c	0.80b	0.77b	0.93a	1.03a
Fruit Shape Index Internal	0.93d	1.07 cd	1.27c	1.93b	2.83a
Dry Matter (%)	6.10d	7.10c	7.82b	7.91b	10.03a
Total Sugars (%)	1.20d	1.40c	1.60b	1.60b	2.07a
Total Phenols (mg/100 g)	153.77e	165.13d	172.03c	174.87b	225.70a
Anthocyanin Content (mg/100 g)	0.30d	0.87c	1.00b	1.00b	1.73a
Nematode Reaction Scale	Susceptible	Susceptible	Susceptible	Susceptible	Moderately Resistance

‡ Values in a column followed by the same letter are not significantly different, p ≤ 0.05, LSD.

## 4 Discussion

Nematodes possess a diverse host range and are among the leading causes of yield loss in eggplant worldwide. Furthermore, yield losses because of plant-parasitic nematodes are prone to surge in the future because of harvest methods and climate change intensification (Abd-Elgawad and Askary, 2015, Sikora et al., 2018, Sivasubramaniam et al., 2020). Recently, biological control has turned into a cost-effective and eco-friendly method for handling nematodes and increasing harvest yields (Hussain et al., 2020, Nega, 2014). In this regard, AMF is among the most eco-friendly forms of controlling plant parasites and boosting crop yields. AMF aids plant development in combination with PSB due to improved nutrient absorption in return for a multitude of photosynthetic carbon (Parray et al., 2019). Additionally, their mixture effectively alleviates abiotic and biotic-induced plant stress, like RKNs. It is worth mentioning that plants with more effective rhizosphere nutritional supplies could withstand considerable growth of parasite nematode infestations (Schouteden et al., 2015).

Furthermore, fungal hyphae are much smaller as compared to the start and could penetrate narrow pores to take in extra nutrition (Püschel et al., 2020). Similar to our results, as we have noticed the overall effect of AMF and PSB on eggplant morphological and biochemical traits, several recent studies also point towards the usefulness of AMF inoculum for eggplant (Chaturvedi et al., 2018, González-González et al., 2020, Sabatino et al., 2020). AMF enhances fruit quality by boosting growth and is considered a vital part of agricultural production. It adds drastically to the sustainability of farming systems if correctly handled (Mahanty et al., 2017, Parnell et al., 2016). Further, nematodes and AMF are recognized crucial co-inhabitants of plant roots. *M. javanica* and AMFs compete over food and space (Schouteden et al., 2015). In such scenario, PSB and AMF not only induce nematode resistance, enhancing harvest yield and quality, but also develop and incorporate green crop management.

Host resistance or responsiveness by AMF may be considered a promising option. *M. javanica* generally harms growth and development, while AMF can improve host resistance and increase adversaries by slowing down nematode advancement (Hol and Cook, 2005). Parasitism dynamics can coordinate nematode communities, which will impact even the characteristics of AMF encounters. Many root cells remain necrotic after feeding nematodes, while others develop cells for specialized feeding constructions (Bécard, 2017, Skiada, 2019). Because of the variety of species participating in belowground activities, determining the exact consequences of different classes is difficult. Nevertheless, our findings suggest that AMF may regulate root herbivores associated with eggplant. To fully comprehend nematode control in natural systems, further research is needed into nematode antagonists' function, the impact of AMF in other nematode genera, and the implications of this interaction for nematode competition. Nematode colonization and replication were further reduced after plants were pre-inoculated with AMF. Since ectoparasite nematodes have a significant effect on AMF compared to *M. javanica*, they are much more protective compared to sedentary nematodes with sophisticated and advanced feeding methods (Ravichandra, 2014). Similar trends are also noticed for plants tolerance to abiotic stress (Ahmad et al., 2010; Ahmad et al., 2019, Ali et al., 2019).

The significant effects of rhizobacteria on eggplant growth can also be attributed to rise in plant immunity to nematodes. These bacteria survive in the soil around plants, provide growth-promoting materials and boost overall plant immunity. This is achieved by maintaining the supply of nutrients from the plant roots, along with the synthesis and control of phytohormones, contributing to an increase in biomass. Our results are in agreement with Siddiqui et al. (2005) as the author revealed PSB ability to stimulate and produce defensive compounds. PSB modulate biological and physical properties of soil that enhance plant growth parameters, i.e. root and shoot length and weights compared to nematode-treated plants. In this direction, Soliman et al. (2011) reported that *A. chroococcum* and *A. brasilense* are useful in controlling RNK infestation. Likewise, recorded AMF is effectively added to optimize reduction in egg mass/root, egg/egg mass, nematode, and root-knot indices in cultivated eggplant compared to control (Elkelany et al., 2020). Some other disparities in broad feeding categories are crucial to establish AMF's interaction attributes (Gough et al., 2020). Nematodes can have an impact on AMF by destroying cells that support AMF feeding. Several root cells stay necrotic until they are nourished while other nematodes induce the cells to build specialized feeding structures (Joseph, 2013, Ravichandra, 2014, Kaya et al., 2020). A far more subtle way that nematodes could influence AMF is the triggered plant response that produces far less susceptible/adaptable cells to AMF. In these interactions, co-evolution is assumed to have culminated in specific identification processes (gene-for-gene interactions). They are likely to be correlated with the non-induction or suppression of typical plant defense responses (Corradi and Bonfante, 2012, Kohli et al., 2019). These undoubtedly lead to the abolition or failure of big plant protective responses to all the microorganisms ordinarily studied for regulation of cultivating parasites (Smant et al., 2018). In short, there is a need to just find out where the AMF and the nematodes are and, to recognize the potential mechanisms. Therefore, more focus studied should look at the endpoints and try to identify and characterize the critical phases of the engagement activities.

## 5 Conclusions

Eggplant cultivation is becoming popular because of its outstanding nutritional qualities. But several biotic variables impede its successful production. The RKN is one of the most destructive agents for eggplant, and its treatment is far more complicated than other pathogens. AMF signifies antagonistic effects on the nematode, and they also improve the eggplant's biochemical or physical properties. AMF inoculation can promote growth and development of the plant, enhance tolerance to abiotic and biotic stress, improve resistance to various pathogens and eventually improve the product quality of eggplants under unfavorable conditions. However, these desired effects depend upon initial selection and optimization of AMF inoculum. Different species of AMF (Gm and Gg, in this study) are known to increase the amount of antioxidant compounds (anthocyanin, total phenols etc.), essential minerals, and fiber content in the plant. Similarly, when applied to eggplant, AMF inoculation improved morphological and biochemical traits under nematode infestation. Significantly, AMF application affected plant growth indirectly, and in some cases, it lowers the demand for harmful chemical pesticides. Thus, the use of AMF and PSB is ideal for sustainable RKN prevention in eggplant.

## Declaration of Competing Interest

The authors declare that they have no known competing financial interests or personal relationships that could have appeared to influence the work reported in this paper.
